# Monthly Summary

**Published:** 1875-07

**Authors:** 


### MONTHLY SUMMARY.

*The Diagnosis of the "Lead-line."*—In chronic lead poisoning a blue line appears on the gums. But this may be simulated by other substances deposited there. Dr. Gras recommends that when we are in doubt whether a given blue line on the gum be due to lead or not, we should excise a fragment of the gum containing the line, with a fine sharp scalpel or the point of a lancet, wash it with a camel's hair pencil, and add a drop of glycerine; if necessary, flatten it out with needles, and examine it under the microscope with a low power. If the line be due to lead, in the midst of the normal tissues of the gum we shall find capillaries injected, filled and obstructed by blackish granules. These capillaries are in loops, or semi-circular, or like double hooks, the outlines varying somewhat according to the section. In very old lead-lines the capillary walls are less evident, and their outlines somewhat indistinct. If a piece of buccal mucous membrane be excised, we should use carmine with glycerine, and a little dilute acetic acid, which shows the mucous papillæ, and the capillary network. He suggests that in fatal lead-colic, the intestinal capillaries and the nerves of the solar plexus should be examined in the same way for lead. It has long since been proposed to examine the lead-line by a simple microscope—or in other words, a one or two inch biconvex lens; when, if in the capillaries, as the true lead-line is, it will be seen clearly to be dotted, and to follow the course of the vessels.—*Med. and Surg, Reporter.*

---

*Cold Powder.*—Dr. George M. Beard states that for a long time, with great success, he has used in breaking up colds the following powder:

Camphor, five parts; dissolve in ether to the consistence of cream; then add carbonate of ammonia, four parts; opium powder, one part; mix and keep in a tightly corked bottle. The dose ranges from three to fifteen grains. In a general way, directions are given to take as mnch as can be held on the finger nail. The powder is to be taken whenever there is a suspicion of having caught cold, and repeated as needed, The Dr. says that it is agreeable to take and singularly efficacious.—*Detroit Review of Medicine and Pharmacy.*

*Lead Poisoning in Lead Workers.*—Dr. Ramskill has given a *resume* of sixty cases of poisoning among white-lead workers, who had been under treatment in the wards of the London Hospital, within the past two years. Of the sixty, thirty-three were males and twenty-seven females. Of the males, the majority were between twenty and thirty years of age, only seven being over forty. The fact that the largest number who were poisoned were under thirty years of age would seem to indicate, he thinks, that the men are unable to work for a long period without suffering. Of the twenty-seven females, three were under twenty, three over forty, and of the remainder, nine were between twenty and thirty, and eleven between thirty and forty. From this it would seem, at first sight, that the women were able to continue at the work longer than the men—but he found, on inquiry, that the latter commenced work earlier in life, and therefore were younger when attacked.

The early or late appearance of poisoning varied with their special employments, the most injurious occupation seeming to be attending to the drying and carrying of the newly dried material to the workhouse.

This newly dried white lead is powdery and easily shaken about, and so may enter the system by the mouth, in respiration, or by becoming intimately mixed with the clothing and infecting the body through contact with the skin.

The treatment at first consisted of removal of the patient from the source of the disease, rest, and in some cases the administration of quinine and sulphuric acid ; in others, sulphate of magnesia, followed by iodide of potassium. Two of the sixty patients died—both being young women and sisters, and in each epileptiform convulsions were features of the case. In one, lead was found in the brain.—*British Medical Journal.*

---

*Thymol an Antiseptic and Antifermentative Substance.*—At the suggestion of Prof. Liebreich, Lewin has investigated the properties of thymol, and has published a part of the results at which he arrived. He states that this substance, the formala of which is C-10 H-14 C-2, is a benzol obtained by distillation from the oil of thyme, and consists of highly aromatic white crystals soluble in 1000 parts of hot water. A solution even as weak as this exhibits all its peculiar properties. He found that saccharine fermentation was wholly prevented by a 1-16th per cent. solution of thymol, while solutions of carbolic and salicylic acid four times as strong were not nearly so efficacious. The thymol solution also at once arrested a fermentation which had already begun. The quantitative determination of the loss of weight by the process of fermentation gave even more striking results. He found that milk treated with the thymol solution did not coagulate till twenty days later than when mixed with the same quantity of simple water, while it remains perfectly sweet and free from mould at the end of five weeks. The same was the case with white of egg after eleven weeks. Putrid pus, when mixed with the thymol solution, lost its fetid smell, and remained in this condition until it dried up. Urine similarly treated did not on an average show signs of decomposition till the end of five weeks. He declares, moreover, that thymol is capable of arresting or preventing the action of putrid pus upon the animal system, and is decidedly deodorizing. The high price of the article he regards as of minor importance, because it may be used in such dilute solutions. When taken into the mouth a 1-10th per cent. solution caused a slightly burning and astringent sensation. When taken into the stomach it appeared to prevent fermentative changes, but not to interfere with digestion.—*Centralblatt f. d. med. Wissensch.*

---

*Case of Abstention from Food.*—The subjoined case reminding us of the "Welsh fasting girl," and Louise Lateau, is communicated to us by Dr. D. R. Silver, of Sidney, Ohio, who vouches for the truth of the report from his own observation. The extract is from a local paper.

"Sarah Brown, daughter of Andrew Brown, of Turtle Creek township, aged about twenty-three years, has been for five years afflicted with an obscure form of nervous disease. For the larger portion of this time she has been confined to her bed. For two years she has been totally blind, and for the same time has been wholly devoid of the power of speech. She lies at the present time helpless as an infant. Her hands are tightly closed, so that if she had muscular power, she could not help herself. At times she has severe pain, cramp, and muscular contractions in various parts of her body. The strangest part of this case, however, remains to be told, At various times during her sickness her appetite has been exceedingly capricious, sometimes for three weeks taking nothing but a little milk. On the first of January of this year she refused to take any food, either solid or liquid, and absolutely continued her fast until the first of March, fifty-nine days in all, and for over fifty days her bowels were in a state of complete constipation. During this time her friends made attempts to induce her to take food or medicine, but without success. Even water having bread soaked in it, and carefully strained, was promptly rejected. Her only ailment was pure water, of which she drank freely. On the sixtieth day she was induced to swallow a little cider, and since that time she has been taking a little solid food. Incredible as this story may appear, this much is certain, that the girl cannot be a malingerer, from the nature of her case."—*Medical and Surgical Reporter.*

